# Genetic and Molecular Control of Floral Organ Identity in Cereals

**DOI:** 10.3390/ijms20112743

**Published:** 2019-06-04

**Authors:** Zulfiqar Ali, Qasim Raza, Rana Muhammad Atif, Usman Aslam, Muhammad Ajmal, Gyuhwa Chung

**Affiliations:** 1 Institute of Plant Breeding and Biotechnology, Muhammad Nawaz Sharif University of Agriculture, Multan 66000, Pakistan; 2 Department of Plant Breeding and Genetics, University of Agriculture, Faisalabad, Pakistan; qasimnazami@gmail.com (Q.R.); dratif@uaf.edu.pk (R.M.A.); wellusman@hotmail.com (U.A.); ajmalrana100@gmail.com (M.A.); 3 Molecular Breeding Laboratory, Division of Plant Breeding and Genetics, Rice Research Institute, Kala Shah Kaku 39020, Pakistan; 4 Centre for Advanced Studies in Agriculture and Food Security, University of Agriculture, Faisalabad 38000, Pakistan; 5 Department of Biotechnology, Chonnam National University, Chonnam 59626, Korea

**Keywords:** ABCDE model, cereals, evolutionary relationships, flower organ identity, floral speciation, MADS-box genes

## Abstract

Grasses represent a major family of monocots comprising mostly cereals. When compared to their eudicot counterparts, cereals show a remarkable morphological diversity. Understanding the molecular basis of floral organ identity and inflorescence development is crucial to gain insight into the grain development for yield improvement purposes in cereals, however, the exact genetic mechanism of floral organogenesis remains elusive due to their complex inflorescence architecture. Extensive molecular analyses of Arabidopsis and other plant genera and species have established the ABCDE floral organ identity model. According to this model, hierarchical combinatorial activities of A, B, C, D, and E classes of homeotic genes regulate the identity of different floral organs with partial conservation and partial diversification between eudicots and cereals. Here, we review the developmental role of A, B, C, D, and E gene classes and explore the recent advances in understanding the floral development and subsequent organ specification in major cereals with reference to model plants. Furthermore, we discuss the evolutionary relationships among known floral organ identity genes. This comparative overview of floral developmental genes and associated regulatory factors, within and between species, will provide a thorough understanding of underlying complex genetic and molecular control of flower development and floral organ identity, which can be helpful to devise innovative strategies for grain yield improvement in cereals.

## 1. Introduction

Cereals are clearly critical for global food security. They provide approximately 60% of human caloric requirement and this figure can even exceed 80% in resource-poor countries [1]. However, the exponential increase in the world population, soaring food prices and constant depletion of arable land resources due to climate change have made it inevitable to develop cereal crops with increased grain yield [2]. Cereals belong to the grass family Poaceae, which is one of the largest groups of monocotyledonous plants, with almost 12,000 species [3]. The grass family is monophyletic and diverged from eudicots approximately 125–150 million years ago [4,5]. Grasses show remarkable diversity in overall plant morphology, physiology, genetics, and ecology compared to their eudicot counterparts [6]. For example, spikelets are characteristic structural units of grass inflorescence, which (depending upon species) show determinate or indeterminate growth. Spikelets are composed of one to several florets but unlike eudicot flowers, these florets possess bract-like structures called lemma, palea, and lodicules, instead of sepals and petals [7,8]. 

The Poaceae family has two important model crop species; rice (*Oryza sativa)* and maize (*Zea mays).* Each has been used to study flower development processes at the molecular level. The genome of rice is exceptionally small compared to other grass species and has been fully sequenced [9,10]. In addition, the rice genome is conducive for effective positional cloning and genetic transformation; making it ideal for developmental biology studies [11,12,13]. Similarly, the genome of maize has also been fully sequenced [14], is amenable to positional cloning, and the species has simple reproductive biology [7,15]. Both of these species show synteny [9], thus the progress in one species has been facilitating the progress in the other species. 

In addition to rice and maize, the Poaceae family also contains *Brachypodium distachyon*, a promising model plant that is anatomically similar to the majority of forage grasses and temperate cereals including wheat (*Triticum aestivum*), the “king of cereals”. *B. distachyon* has a short life cycle and is readily cultivatable. The genome of *B. distachyon* has already been sequenced [16] and it offers a highly efficient genetic transformation system. These qualities make *B. distachyon* suitable for functional genomic studies of grass related traits [17,18,19]. In comparison, wheat is the most important staple crop in temperate zones and a major source of starch, energy and dietary fiber. As an example, bread wheat alone provides 20% of the daily calorie intake in the UK [20]. However, wheat functional genomic studies were limited due to the lack of a quality reference genome sequence and hexaploid nature of the species [21,22]. More recently, a high quality, fully annotated reference genome of hexaploid wheat has been delivered which can accelerate research in wheat developmental biology and genomics assisted breeding [23]. In recent years, significant progress has been made towards understanding the genetic regulation of spike development in Brachypodium, wheat, and barley [24,25,26,27,28,29,30,31]. These studies revealed striking similarities between Brachypodium, wheat, and barley, with highly conserved genetic regulation of inflorescence development in these species. Thus, understanding the molecular control of inflorescence development and floral organ identity in model species will expand our knowledge about the genetic architecture of the spike development in all economically important grasses.

Floral organs control grain development. Previously, a simple yet elegant ABC model of floral organ identity was devised to demonstrate the molecular control of floral development in model plants [32]. This model proposed that combinatorial activities of three homeotic gene classes specify four floral organs i.e., sepal, petal, stamen, and carpel. Class A genes, when expressed alone, produce sepals. The expression of classes A and B together directs petal identity. The expression of classes B and C together regulates stamen identity and the expression of Class C genes alone determines carpel identity. Subsequently, two other floral identity gene classes were identified. Class D genes in Petunia [33] and the redundant class E genes (*SEP1*–4) in Arabidopsis [34,35]. The current model consists of these five classes of floral-homeotic, MADS-box genes (A, B, C, D, and E). The hierarchical combination of these five gene classes thus determines floral organ identity [36].

In higher model plants, especially Arabidopsis and rice, the ABCDE model has helped explain the molecular control of floral organ identity to some extent. This is largely due to their relatively small genome size and the extensive research associated with each of these model species. Analyses of the floral homeotic genes of these species suggest that the same flower organ identity model can be applied to other cereals [37], including Brachypodium, maize, and wheat. This review explores recent advances in rice, maize, Brachypodium, and wheat floral development and subsequent organ specification, with reference to the model plant Arabidopsis. Plethora of studies revealed novel regulatory factors and pathways that contribute to the unique morphology of the grasses. However, the vast array of functions performed by floral homeotic genes and the large body of literature devoted to this subject makes it difficult to comprehensively review all aspects of the genetic control of floral development. Here, we tried to review the comparisons of floral development genes, within and between species that will expand our understanding of the complex molecular genetic control of floral development and flower organ identity especially in grasses.

## 2. Inflorescence Morphology and Development

**Figure 1:**
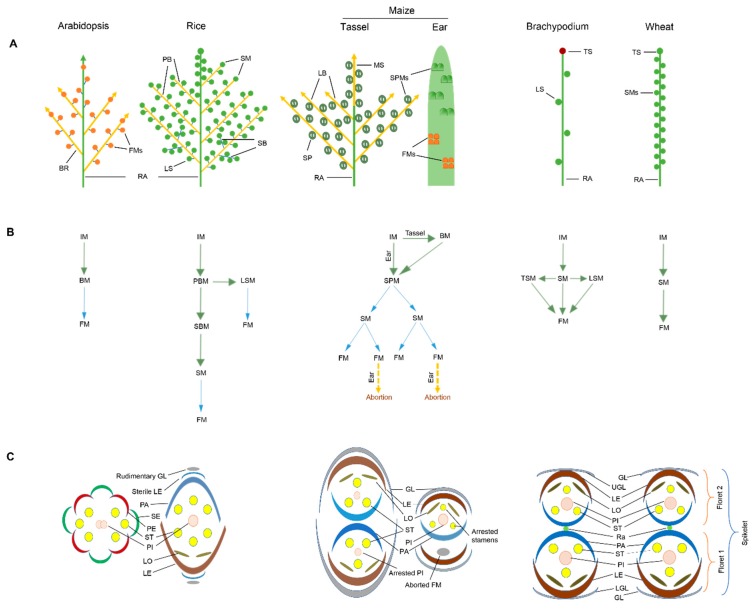
Graphical representation of inflorescences, phase transition and transverse flowers. (**A**) Structural configuration of inflorescence in Arabidopsis, rice, maize, Brachypodium and wheat. Color codes. Green line: rachis; yellow line: primary branch; blue line: secondary branch; green circles: spikelet/spikelet pair meristems; maroon circle/oval: terminal spikelet; orange circle: floral meristems. (**B**) Regulation of meristem transition in Arabidopsis, rice, maize, Brachypodium, and wheat. Green arrow: multiple meristems formation; blue arrow: single meristem formation; orange dashed arrow: abortion of floral meristems. (**C**) Schematic representation of transverse spikelet/flower. Color codes: Blue: palea; dark orange: lemma; gold: lodicules; green: sepal; green circle: rachis; gray: glume; pink: pistil; red: petal; yellow: stamen. Abbreviations: BR: branch; BM: branch meristem; FM: floral meristem; GL: glume; IM: inflorescence meristem; LE: lemma; LO: lodicule; LS: lateral spikelet; LSM: lateral spikelet meristem; PA: palea; PB: primary branch; PE: petal; PI: pistil; PBM: primary branch meristem; RA: rachis; Ra: rachilla; SB: secondary branch; SE: sepal; SBM: secondary branch meristem; SM: spikelet meristem: SPM: spikelet pair meristem; ST: stamen; TS: terminal spikelet.

The grass family includes several agriculturally and economically important species including rice, wheat, maize, sorghum, and barley. Developmental and genetic pathways controlling the shape of inflorescence architecture and development in these important crops have been reviewed briefly [27,28,38,39,40]. All grass inflorescences have a characteristic basal structural unit, the spikelet, composed of one to several florets depending upon the species [6]. These florets are surrounded by bract-like structures known as glumes. Most grass species possess unique inflorescence organization and structure distinct from eudicots and even from other monocots [41]. For example, Arabidopsis bears indeterminate inflorescence with several branched flowers. The grasses like Brachypodium, Hordeum, Secale, and Triticum inflorescences carry sessile spikelets on the rachis. In contrast, Avena, Echinochloa, Oryza, Panicum, Setaria, and Sorghum bear long branched inflorescence where spikelets are pedunculate [42] (Figure 1A). Moreover, the Arabidopsis inflorescence meristem normally differentiates only into branch meristem and floral meristem whereas several specialized axillary meristems are formed in grass spikes [40] (Figure 1B). Unlike their eudicot counterparts, the grass florets possess bract-like structures; lemma, palea, and lodicules in place of sepals and petals [8].

Among cereals, rice exhibits distinct inflorescence morphology compared to that of Brachypodium and wheat [24,38,40,43]. The spikelet is the basal structural unit in these three grass species. The rice inflorescence is relatively complex and comprised of long stalked panicles in which primary branches are directly attached to the main axis (rachis) that further produce secondary branches, lateral spikelets and terminal spikelets [38]. By contrast, there exists only one rachis in Brachypodium and wheat that directly bears the spikelets in an alternating configuration [24,28]. Spikelets in these species also bear rudimentary glumes and floret primordia. In rice, the single spikelet can produce only a single floret [44], whereas the wheat spikelet contains several florets and normally four or five of these reach anthesis [45]. Unlike wheat and rice, the inflorescence of Brachypodium carries only two or three lateral spikelets and a single terminal spikelet [24]. Each spikelet contains ~11 florets, arranged in a distichous phyllotaxy around central axis. Overall, the organization and structure of floral organs are conserved among rice, Brachypodium, and wheat, with the exception of three additional stamens within a floret in rice [24,28,38] (Figure 1C). The grass floret contains lemma, palea, lodicules, stamens, and pistils. The pistil is comprised of three fused carpels which surround a single ovule. The apical region of the pistil bifurcates with feathery stigmas. Morphological analysis suggested that lodicules are homologues of petals [46], which together with the lemma and palea are unique to grasses.

Inflorescence development is regulated by several types of meristems [44,47] and starts with the transition of the shoot apical meristem (SAM) into an inflorescence meristem (IM). In Brachypodium and wheat, the IM directly generates the spikelet meristem (SM) [24,28,43], while in rice the IM generates the primary branch meristem (pBM) followed by the secondary branch meristem (sBM) which then finally configure the spikelet meristems (SMs) [40] (Figure 1B). The SMs generate floral meristems (FMs), which subsequently determine floral organ identity. All grasses show indeterminate growth starting from SAM to just before SM determinacy. However, the SM to FM transition is determinate and critical [48] as it is the final phase at which the meristem activity stops. By this stage, stem cells are believed to exhaust all their energy due to continuous formation of floral organs and floret primordia [47]. 

In contrast to Brachypodium, wheat, and rice, maize is a monoecious crop in which male and female organs occur separately on the same plant. The male inflorescence at the shoot apex is known as tassel that bears paired spikelets while the female inflorescence occurs in the leaf axil which is termed as ear [40]. Male IM produce long indeterminate branches which further differentiate into short secondary branches that bear spikelet pair meristems (SPMs). Each SPM initiate two SMs, which in turn produce two FMs each (Figure 1B). The female inflorescence (ear) is produced on the main stalk, hence SPMs are directly attached to the main stem. SPMs are transient and bear a pair of SMs. SMs are also transient which in turn produce two FMs. Each spikelet bears two staminate flowers called florets and only one of these florets produces a fertile flower. Flowers further develop into different floral organs such as lemma, palea, lodicules, stamens, and carpels. Apart from shapes and position of male and female inflorescences, the arrest of stamen formation in ear florets and of pistil formation in tassel florets makes it easy to distinguish male and female inflorescences [49]. Over the last two decades, several genetic factors involved in flower development have been identified which mainly function as trans-regulatory elements. Here, we will discuss the latest knowledge about the association of MADS-box- and non-MADS-box-related gene families with inflorescence development and floral organ identity in grasses.

## 3. Role of MADS-Box Transcription Factors in Floral Organ Identity

**Figure 2:**
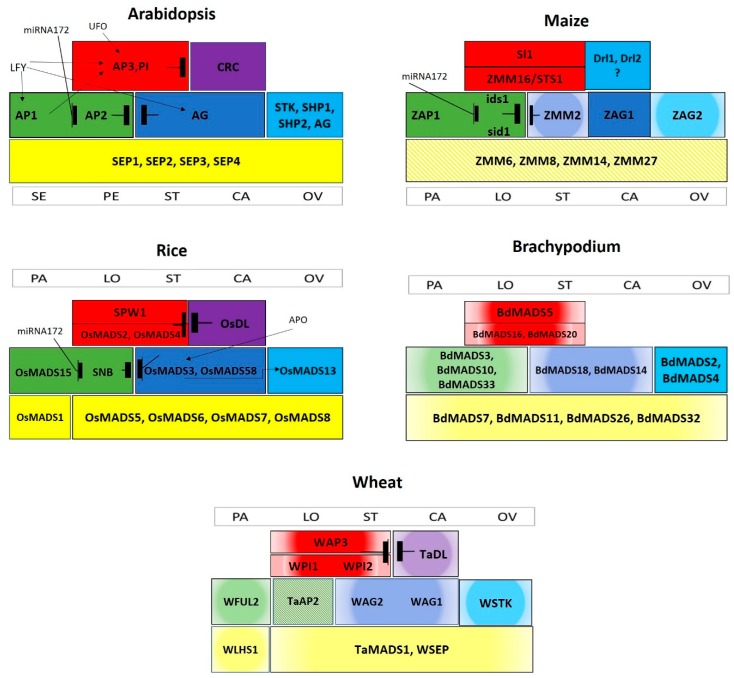
ABCDE models of floral organ identity. Revised floral organ identity models in Arabidopsis, rice, maize, Brachypodium, and wheat. Class (A)-genes indicated in green, class B in red, class C in dark blue, class D in light blue, class E yellow, and non-MADS in purple. Solid colors show functional data, color gradients represent expression analysis data, while color patterns indicate hypothesized functions. Antagonistic interactions are indicated with barred lines, black arrows illustrate positive regulation of the corresponding genes, and a comma symbolizes duplicated gene interaction. Abbreviations: CA: Carpel; LO: Lodicule; OV: Ovule; PA: Palea; PE: Petal; SE: Sepal; ST: Stamen. For gene abbreviations see text.

MADS-box transcription factors are involved in various biological processes and have been identified in almost all groups of eukaryotes. The name MADS was derived from combining the names of *MINICHROMOSOME MAINTENANCE 1* of *Saccharomyces cerevisiae*, *AGAMOUS* of *Arabidopsis thaliana*, *DEFICIENS* of *Antirrhinum majus*, and *SERUM RESPONSE FACTOR* of *Homo sapiens* [50]. All MADS-box TFs have a highly conserved ~60 amino acids long DNA binding MADS domain at the N-terminal region which binds to CArG boxes on DNA [51]. Flowering plant genomes contain approximately 100 MADS-box genes, which are further categorized into M-type and MIKC-type MADS genes [52]. Only a few M-type MADS are functionally characterized so far [53], however, plant MIKC-type MADS-box genes have been extensively studied [54]. In plants, the diversification of MADS-box genes is closely linked to the evolution of important organs, such as seeds, flowers, and fruits [55]. Moreover, morphological variations in inflorescence of grass family are closely associated with changes in copy number, expression patterns, and interactions between MIKC-type MADS-box genes [56]. In flowering plants, combinatorial activities of the five classes of MIKC-type MADS-domain genes define floral organ identity. According to the Arabidopsis “floral quartet model”, sepals are specified by class A and E genes in the first whorl; petals by class A, B, and E genes in the second whorl; stamens by class B, C, and E genes in the third whorl; and carpels by class C, and E genes in the fourth whorl [54]. The ovule identity gene *FLORAL BINDING PROTEIN 11* (*FBP11*) was first identified and functionally characterized in Petunia and classified as D class gene [33]. In Arabidopsis, ovule identity is controlled by *AGAMOUS* subfamily member *SEEDSTICK* (*STK*) [57]. Functional divergence, duplication, and evolutionary relationships among these five classes of homeotic genes, identified in Arabidopsis and major cereals, are summarized in Table 1. Modified ABCDE models showing the complex genetic interaction of MADS-box TFs’ and other important regulators in Arabidopsis and cereals are illustrated in Figure 2.

### 3.1. Class A Homeotic Genes

In Arabidopsis, class A genes include *APETALA 1* and *2* (*AP1* and *AP2*), of which only *AP1* encodes a MADS-box TF. *AP1* is expressed only in sepals and petals (two outer whorls) and has an additional role in floral meristem determinacy [61]. Similar expression and functional patterns have been reported in Antirrhinum class-(A) ortholog [127]. In eudicots, class (A)-functions are defined by *AP1*, *CAULIFLOWER (CAL)*, and *FRUITFULL (FUL)* genes, whereas in monocots only *FUL*-like genes are present which are associated with class (A)-function [128]. Recently, Wu et al. [65] demonstrated that grass-specific *FUL*-like genes are required to specify palea and lodicule identities in addition to their function of specifying meristem identity. Similar results were reported for rice and wheat, wherein *AP1* clade genes together with class E *SEPALLATA* (*SEP*) genes were shown to participate in the transition from SAM to IM [112,129]. 

The *AP1* homologs identified in rice include *OsMADS14*, *OsMADS15*, *OsMADS18*, and *OsMADS20*, all of which belong to the *FRUITFULL (FUL)* lineage [59]. Ectopic expression of *OsMADS14* in rice suggests its involvement in floral meristem control to promote flowering. On the other hand, loss-of-function loss-of-function mutations in *Osmads15* indicate the role of *AP1* in palea formation with no effect on lodicule development [130]. A more recent study in rice employing both single and double mutants of *OsMADS14* and *OsMADS15* [65] provided strong evidence that rice *AP1/FUL*-like genes are essential for specifying lemma/palea and lodicule identities during the floral development process. Because lemma and palea are considered homologous structures to sepals and petals of eudicots, respectively, therefore, it is possible that *AP1/FUL*-like genes are independently recruited to fulfil the function of class A genes in grass species. Maize orthologs of *AP1* include *Zea mays APETALA1* (*ZAP1*), *Zea mays MADS4*, and *15* (*ZMM4*, *ZMM15*) [62]. Phylogenetic analysis showed that *ZAP1* is an ortholog of *OsMADS15* [131]. Northern blot analysis demonstrated that *ZAP1* was expressed in the lemma/palea and lodicules, but not in stamens and pistils [62]. These results suggest that *ZAP1* is a putative class-(A) gene with a possible repressive interaction with class C genes. *ZMM4* and *ZMM15* are orthologs of *OsMADS14* and *ZMM4* and have been reported to be involved in inflorescence development and floral induction [58], which is consistent with the function of *AP1* homologs from Arabidopsis and rice. 

The Brachypodium genome contains at least four (A)-class genes, *BdMADS3*, *10*, *31*, and *33*, which are orthologs of *OsMADS18*, *OsMADS15*, *OsMADS20*, and *OsMADS14*, respectively. *BdMADS3*, *10*, and *33* were observed to be strongly expressed in the lemma and palea, but not in lodicules and stamens with the exception of *BdMADS3* that also strongly expressed in stamens [31]. *BdMADS31* was absent in all floral organs but was weakly expressed in leaves similar to the expression pattern of Arabidopsis and rice orthologs [132,133]. These expression pattern studies suggest the involvement of *BdMADS3*, *10*, and *33* in (A)-class performance; however, further functional analyses are required to confirm their regulatory roles in floral organ identity.

**Figure 3:**
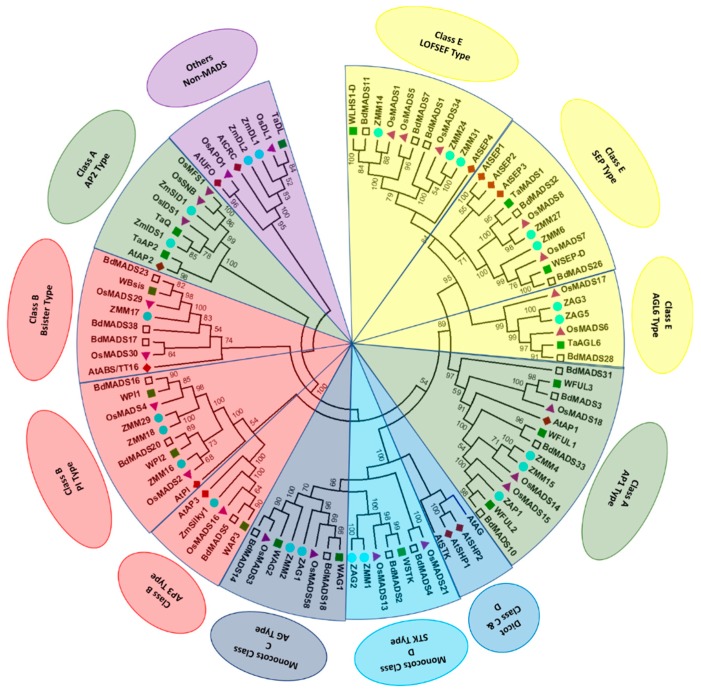
Evolutionary relationships among MADS-box genes. Phylogenetic tree constructed from the deduced amino acid sequences of Arabidopsis, Brachypodium, maize, rice, and wheat genes obtained from NCBI database. (Sequence ID information can be seen in supplementary file) The tree was inferred after amino acid sequence alignment by Clustal Omega [137], using the neighbor-joining method [138] and visualized in topology-only mode. Only bootstrap values >50%, as calculated from 100 replicates, are shown. Phylogenetic analysis was conducted in MEGA version 6 [139]. Markers: Diamond: Arabidopsis; triangle: rice; circle: maize; green filled square: wheat; hollow square: Brachypodium genes.

Wheat has five *FUL*-like paralogs including *WFUL1*/*VERNALIZATION1* (*VRN1*), *WFUL2*, *WFUL3*, *TaAGL10*, and *TaAGL25* [117,128]. Phylogenetic analysis showed that these are the orthologs of *OsMADS14*, *OsMADS15*, and *OsMADS18* [60], an observation consistent with the current phylogenetic tree (Figure 3). Previously it was thought that *WFUL1* had no (A)-class function and was only involved in the transition from the vegetative to reproductive phase [63,134], but recent studies suggest that *VRN1/WFUL1* is expressed in leaves and the shoot apex, where it is required for the long-day flowering response and inflorescence meristem identity [64,134,135]. *ODDSOC2* is a MADS-box TF and downstream target of *VRN1* that functions to repress flowering and has been observed to be downregulated in plants with active *VRN1* alleles and vernalization [136]. Another study reported that *WFUL1* and *WFUL3* are expressed in all floral organs with limited or no expression of *WFUL2* in stamens and pistils [60], suggesting that *WFUL2* has diversified functions in outer (palea and lodicule) and inner (stamen and pistil) floral whorls. Yeast two-hybrid and yeast three-hybrid analyses demonstrated that *WFUL2* interacts with the B and E classes of MADS-box genes [60]. These findings in combination with the expression pattern analysis illustrate that *WFUL2* has a major role in lemma/palea and lodicule identities in wheat florets. It is noteworthy that *WFUL1/VRN1* has a more important role in leaf development indicating functional diversification between wheat *FUL*-like genes. Similarly, functional diversification between rice *FUL1* (*OsMADS14*) and *FUL2* (*OsMADS15*) has been observed. Single mutant of *OsMADS14* showed lower seed setting, but no floret-specific mutant phenotype could be observed when grown under natural field conditions. However, under greenhouse conditions the mutant plants had small paleae and showed the homeotic transformation from lodicules to stamen-like organs. Whereas paddy field-grown *osmads15* plants showed 45% smaller paleae, without affecting the organ identity. However, greenhouse-grown *osmads15* plants had elongated empty glumes and 100% reduced paleae. Additionally, *osmads15* plants showed no homeotic transformation of inner three floral organs under both growing conditions [65].

All angiosperms contain *AP2* TFs, which, in addition to their role in the regulation of floral development, are implicated in primary and secondary metabolism, growth and development, and response to stress [140]. In Arabidopsis, *AP2* is required for the establishment of floral meristems, floral organ identity, and regulation of floral homeotic gene expression [70]. In rice, two *AP2*-like genes—*INDETERMINATE SPIKELET1* (*IDS1*) and *SUPERNUMERARY BRACT* (*SNB*)—synergistically control lodicule development [72]. Another *AP2*-like gene, named *FRIZZLE PANICLE* (*FZP*), prevents the formation of axillary meristem in rice but controls the spikelet meristem identity [71]. *FZP* has also been observed to regulate the transition from panicle branching to spikelet formation in rice by repressing *RICE FLORICAULA LEAFY* (*RFL*)/*ABERRANT PANICLE ORGANIZATION2* (*APO2*). In addition, *FZP* overexpression positively regulate B and E class MADS-box genes in floral meristem suggesting its role in floral organ identity [67]. *MULTI-FLORET SPIKELET1* (*MFS1*) is another *AP2*-type gene that positively regulates rice *IDS1* and *SNB* genes [74]. Rice *IDS1* and *SNB* regulate the transition from spikelet meristem to floral meristem [141]. Both of these genes display strong functional resemblance to maize indeterminate spikelet1 (*ids1*) and sister of indeterminate spikelet1 (*sid1*), respectively, which are also required to initiate floral meristems and to control spikelet meristem determinacy [68]. Similar to the function of *AP2* in Arabidopsis, *ids1* and *sid1* negatively regulate class C gene function within the lateral organs of the spikelet. Likewise, maize *BRANCHED SILKLESS1* (*BD1*) encodes an ethylene responsive factor (*ERF*/*AP2*) that regulates the spikelet meristem identity and mutation in *BD1* produces indeterminate floral branching [69]. Like rice *FZP* and maize *BD1*, Brachypodium *MORE SPIKELETS1* (*MOS1*) determines spikelet meristem identity as the *mos1* mutant showed increased number of axillary meristems compared with the wild type [24]. In wheat, Wheat *FZP (WFZP)* controls spikelet meristem identity that drives the formation of supernumerary spikelets by repressing floral meristem formation and differentiation [25]. The regulation of spikelet meristem identity by *AP2-*like genes in rice, maize, Brachypodium, and wheat indicates that their function is conserved among distantly related grass species including agriculturally important crops. In addition, wheat genome also contain *TaQ* and *TaAP2*. The wheat domestication gene (*TaQ*) has a role in inflorescence shape, glume shape, glume tenacity, and spike length [27,75]. Phylogenetic analysis and transcriptional pattern of wheat *TaAP2* revealed its orthologous relationship with barley *HvAP2/Cly1*, which is involved in lodicule identity [73,142]. This observation demonstrates that like rice *AP2*-like orthologs, wheat *TaAP2* might also associated with lodicule identity [72,74], suggesting their functional similarities in grasses.

In recent years, evolutionary conserved micro-RNAs (miRNAs) have been identified and played a crucial role in plant organogenesis. miR172 appears with the evolution in angiosperms and has been identified in Arabidopsis, rice, maize, barley, and wheat. The level of miR172 increases with plant age and its expression is under photoperiodic control [143]. It is an active repressor of all *AP2*-like TFs, which are thought to participate in floral patterning. *AP2* has been demonstrated to bind and repress the expression of miR172b [144]. Early studies reported *AP2* transcripts in all floral organs [70], however recent observations show that *AP2* expression is restricted to sepals and petals compared to that of miR172 that predominantly expressed in inner floral whorls (stamen, carpel, and ovule) [145]. These findings suggest an antagonistic interaction of *AP2* and miR172 in plant developmental transitions. 

In cereals, functionally characterized targets of miR172 include *Zea mays* indeterminate spikelet1 (*ids1*) and sister of indeterminate spikelet1 (*sid1*) [68], *Oryza sativa SUPERNUMERARY BRACT (OsSNB)* [146], and *Hordeum vulgare Cleistogamy1 (Cly1)* [142]. Wheat domestication gene *TaQ* is also a target of miR172, however it is not clear if miR172 mediated regulation has a role in domestication [147]. These investigations provide new insights into the ancient role of miRNAs about floral organ regulation in cereals.

### 3.2. Class B Homeotic Genes

Arabidopsis class B homeotic genes include *AP3* and *PISTILLATA* (*PI*) that are required for petal and stamen identities. Single mutants of these genes caused conversion of petals to sepals in the second floral whorl and stamens to carpels in the third floral whorl [78,81]. Rice has two orthologs of *PI*: *OsMADS2* and *OsMADS4* [148]. RNAi suppression of *OsMADS2* showed homeotic changes in lodicules with no effect on stamens [83], whereas RNAi suppression of *OsMADS4* showed no alteration in these floral organs [86]. Interestingly, simultaneous mutations in both genes caused the conversion of lodicules and stamens into palea and carpel-like structures respectively. These observations suggest an equal role for both genes in stamen development, with *OsMADS2* more important in lodicule identity. Similarly, maize contains three orthologs of Arabidopsis *PI*; *Zea mays MADS16*, *18*, and *29* (*ZMM16*, *ZMM18*, and *ZMM29*). Mutation in *ZMM16* produced a *Sterile Tassel Silky Ear1* (*STS1*) phenotype in which lodicules transformed into palea-like and stamens into carpel-like structures [85]. Phylogenetic analysis showed that *ZMM16* as an ortholog of *OsMADS2* while *ZMM18* and *ZMM29* are orthologous to *OsMADS4*. Recently, a study reported that *ZMM16*/*STS1* (together with its paralogs *ZMM18* and *ZMM29*) forms obligate heterodimers with maize *SILKY1 (Sl1)* and specifies organ identity in second and third floral whorls [149]. Interestingly, RNAi knockdowns of *ZMM18* and *ZMM29* showed no detectable floret phenotype, indicating that *STS1* can compensate *ZMM18*/*29* reduction, but *ZMM18*/29 cannot compensate for *STS1* reduction. With this evidence, it is possible to speculate a role for maize *AP3*/*PI*-like genes.

The sole ortholog of *AP3* in rice; *OsMADS16*/*SUPERWOMAN1* (*SPW1)* has been observed to interact with rice *PI*-like genes. *OsMADS16* knockdown mutant showed homeotic conversion of lodicules and stamens to palea and carpel-like structures, respectively, similar to *PI*-types [80]. Similarly, the loss-of-function mutant of maize *SILKY1* (*AP3* ortholog) showed alterations in lodicules and stamens [76]. As lodicules represent second whorl (petals), their transformation into palea-like structures support the hypothesis that petals of eudicots are likely to be modified into lodicules in grasses. Furthermore, Arabidopsis and maize class B genes showed similar biochemical activities in vivo and in vitro [85]. Collectively, these findings suggest that the function of class B genes is somewhat conserved between grasses and eudicots. 

The Brachypodium genome contains three B class genes: *BdMADS5*, *16*, and *20*. *BdMADS5* is an ortholog of Arabidopsis *AP3* and rice *OsMADS16*. Similarly, *BdMADS16* and *BdMADS20* are orthologs of *OsMADS4* and *OsMADS2*, respectively, and are clustered with Arabidopsis *PI* [31]. Strong expression of Brachypodium B class genes was detected in lodicules and stamens, with *BdMADS16* expressed in carpels as well. However, transcript abundance of all B class genes was very low in the lemma and palea in Brachypodium similar to those of Arabidopsis and rice B class genes [132,133]. Although their expression patterns suggest that *BdMADS5*, *16*, and *20* have conserved roles in lodicule and stamen identity, functional analyses of these genes remain to be conducted to confirm these hypotheses.

*WAP3*, also called *TaAP3* is a wheat ortholog of Arabidopsis *AP3*, which is encoded by two highly homeologous genes: *TaMADS#51* and *TaMADS#82* [82,131]. Northern blot analysis revealed that *WAP3* expression was restricted to young spikes during floral development and possibly associated with the induction of pistillody (homeotic conversion of stamens into carpel-like structures) [79]. *WAP3* is also involved in the homeotic transformation of lodicules and stamens into palea and pistil-like structures, respectively [77]. Wheat genome also contains two *PI*-like genes: *WPI1* and *WPI2*. Phylogenetic analysis revealed close orthologous relationships of *WPI1* with *OsMADS4*, and that of *WPI2* with *OsMADS2*. Similar to *WAP3*, wheat *PI*-type genes were reported to be involved in lodicule and stamen development and their homeotic transformation into palea and pistil-like structures. Hama et al. [77] reported that *WAP3* and *WPI* were highly expressed in the primordia of lodicules and stamens. Low expression patterns of wheat B class genes were detected in pistil-like stamens of an alloplasmic wheat line having the *Aegilops crassa* Boiss. cytoplasm and lacking the *Rfd1* gene, indicating that these genes gradually disappear from the fourth whorl (carpel/pistil) just like Arabidopsis *PI* [81]. These observations strongly suggest that wheat class B genes are associated with the induction of pistillody, a direct consequence of changes in copy number and expression of *WAP3* and *WPI’s* in third and fourth whorls confirming that *WAP3* and *WPI’s* exhibit class B functions.

*BSISTER* genes, closely related to class B MADS-box genes, have been identified through phylogenetic studies. Members of this subfamily regulate female reproductive organs and seed development [150]. All *BSISTER* MADS-box genes investigated to date are expressed during early ovule development indicating that these genes may be required for ovule identity. Arabidopsis has two *BSISTER* genes—*ARABIDOPSIS BSISTER (ABS)/TRANSPARENT TESTA16* (*TT16*) and *GORDITA* (*GOA*)—both expressed in mature ovules [88,89]. Yang et al. [91] has functionally characterized the rice *BSISTER* MADS-box gene; *OsMADS29*. His findings demonstrate that *OsMADS29* expressed only in floral but not vegetative organs. Another study involving RT-PCR revealed that *OsMADS29* expressed in ovules, consistent with previously reported patterns for wheat *BSISTER* (*WB_sis_)* and maize *ZMM17* [87,90]. However, knock-down of *OsMADS29* by double-stranded RNA-mediated interference (RNAi) resulted in shriveled and/or aborted seeds [91], suggesting that *OsMADS29* also has important functions in seed development of rice by regulating cell degeneration of maternal tissues. Furthermore, Arabidopsis and rice *BSISTER* and D-class genes show overlapping expression patterns [151]. More recently, Schilling et al. [84] investigated another *BSISTER* gene (*OsMADS30*) in rice. This gene was weakly expressed in ovules. Further, the plants carrying a T-DNA insertion in *OsMADS30* showed no aberrant phenotype, indicating that this gene is either not required for ovule specification or its function is obscured by another class D gene (*OsMADS21*). Brachypodium also contains three *BSISTER* genes: *BdMADS17*, *23*, and *38*. Weak expression of *BdMADS17* and *BdMADS23* was detected in palea but absent in ovules. However, *BdMADS38* was weakly expressed in stamens only [31]. Altogether, these results suggest that *BSISTER* genes do not possess a strict function, instead of play overlapping roles in whole reproductive ontogeny. 

### 3.3. Class C and D homeotic genes

It is believed that during the divergence of angiosperm and gymnosperm lineages, an ancient duplication resulted in the class C origin, including all stamen and carpel identity genes and class D or ovule specification genes [98,99,152]. This type of classification is reported in several phylogenetic studies [30,31,131,153], and has therefore been adopted in this review. Arabidopsis has three class C homeotic genes; *AGAMOUS* (*AG*) and *SHATTERPROOF1* and *2* (*SHP1* and *SHP2*). Arabidopsis typical class C gene *AG* specifies stamen (third whorl) and carpel (fourth whorl) identities and has an additional role in floral meristem determinacy [93]. In the absence of *AG* activity, class (A)-gene function expands to the 3rd and 4th whorls [32,154], which suggests antagonistic interaction between these two classes of homeotic genes. The additional C class genes of Arabidopsis, *SHP1* and *SHP2*, are required for carpel and fruit dehiscence zone specifications [57,155]. Like grass class B genes, the function of class C genes are also diversified in grasses due to events of duplication and subfunctionalization of these genes during evolution. Rice has two duplicated class C genes; *OsMADS3* and *OsMADS58*. Yamaguchi et al. [98] investigated single mutants of rice class C genes and reported interaction with the class D gene *OsMADS13*, regulating floral meristem determinacy with redundant mediation of class C gene functions. Mutant and transgenic analyses showed that *OsMADS58* regulates floral determinacy with minor effects on carpel identity, while *OsMADS3* predominately regulates stamen identity and prevents lodicule development with minor effects on floral determinacy. As floral determinacy is defined by class (A)-genes, *OsMADS58* probably has reduced *AG* activity in the third and fourth whorls compared to *OsMADS3*. Furthermore, the rice class B gene *OsMADS16* interacts with class C genes to suppress indeterminate growth within floral meristems [156]. These findings indicate that class C genes play a dominant role in stamen and carpel identity, with a minor role in floral meristem determinacy and possible antagonistic interaction between A and C class genes. A study conducted by Dreni et al. [92] demonstrated redundant mediation of the class C associated functions by *OsMADS3* and *OsMADS58*. He also observed strong defects in stamens and carpels of *osmads3* flowers, whereas most of the *osmads58* flowers were indistinguishable from wild type flowers. The contribution of *OsMADS3* in specifying C-function seems to be more important when compared with *OsMADS58*, consistent with the reports of Yamaguchi et al. [98]. The double mutants of *osmads3* and *osmads58* were corresponding to the *ag* mutant of Arabidopsis with some differences between their phenotypes. The *osmads3* and *osmads58* mutants showed homeotic conversion of stamens and carpels into lodicule and palea-like structures, respectively. Dreni et al. [92] also reported FM determinacy by *AG* subfamily genes. Out of four *AG* subfamily genes in rice, three (*OsMADS3*, *OsMADS13*, and *OsMADS58*) redundantly regulated the FM determinacy. All the three possible double mutant combinations (*osmads3* and *osmads58*, *osmads3* and *osmads13*, and *osmads13* and *o*s*mads58*) resulted in an enhanced FM indeterminacy. 

Maize has three class C genes: *Zea mays AGAMOUS1* (*ZAG1*), *ZMMS2*, and *ZMM23* [152,153]. Like rice class C genes, *ZAG1* and *ZMM2* both have functional diversification as these are orthologs of *OsMADS58* and *OsMADS3*, respectively. Expression analysis detected *ZAG1* transcript abundance in early stamen and carpel primordia with partial floral meristem determinacy [97]. However, a later study with *ZAG1* mutants demonstrated a loss of floral meristem determinacy with little change in stamen and carpel identity [96]. *ZMM2* transcripts were expressed in stamens and carpels, while stronger expression patterns were detected in stamens only, suggesting an involement in stamen and carpel development. Although, *ZMM2* mutants have not been identified, these observations indicate overlapping but nonidentical activities for both maize C class genes.

Like rice, Brachypodium also has two C class genes—*BdMADS14* and *18*—that show high sequence similarity with *OsMADS3* and *OsMADS58*, respectively [31]. Strong expression of *BdMADS18* was detected in stamens and carpels, whereas *BdMADS14* was weakly expressed in stamens only. In contrast to their rice homologs, where *OsMADS3* and *OsMADS58* have important roles in floral organ identity [98]; the gene *BdMADS18* appears to have a more dominant role in stamen and carpel identity. Similarly, wheat also has two orthologs of *AG*; wheat *AGAMOUS-1* and *2* (*WAG-1* and *WAG-2*) [94]. However, unlike rice and maize orthologs, these have possible roles in ectopic ovule formation and the conversion of stamens into pistil-like structures. Meguro et al. [95] reported that *WAG* transcription levels were low in young spikes but increased during later stages of spike development and were highest between the booting and spike emergence stages. *WAG* was expressed in both reproductive and non-reproductive parts of the flower with an extra transcript of *WAG* detected in the pistillody line. These observations suggest that *WAG* is associated with pistillody induction. Loss-of-function analysis of *WAG* genes would further elucidate their role in stamen and carpel identity. Other names for *WAG-1* and *WAG-2* are *TaAG1* and *TaAG2*/*TaAGL39*, respectively [117,131]. Phylogenetic analysis showed that rice class C genes, *OsMADS58* and *OsMADS3*, are orthologous to *WAG-1* and *WAG-2*, respectively (Figure 3). In conclusion, both class B and C genes in wheat appear to have a role in the induction of pistillody [77,79,95]. 

Previous studies demonstrated that class D is a more specialized version of class C and define ovule identity [37,153]. Class D genes were first identified in Petunia as *FLORAL BINDINGPROTEIN 7* and *11* (*FBP7, FBP11*). Their cosuppression transforms ovules into carpelloid structures [157]. Overexpression of *FBP11* results in ectopic ovules on sepals and petals [33] indicating its function in ovule identity. In Arabidopsis, class D gene functions are specified by *SEEDSTICK* (*STK*). Biochemically, STK protein interacts with class C (AG, SHP1 and SHP2) and class E proteins to define ovule identity [101]. Triple mutants of *STK*, *SHP1*, and *SHP2* transform ovules into carpelloid structures [57] confirming that Class D genes specify ovule identity in Arabidopsis. Phylogenetic analysis clustered *STK* and *SHP*s into single clade (Figure 3). The functional divergence between *STK* and *SHP* paralogous genes may arise due to diversification in their DNA binding site motifs or through alterations in their tissue-specific expression levels [158]. Rice contains two orthologs of *STK*: *OsMADS13* and *OsMADS2*1 [99]. Expression analysis, loss-of-function and protein–protein interaction studies suggest that *OsMADS13* is involved in ovule identity [99,100,102,103]. Moreover, *OsMADS13* acts synergistically with *OsMADS3* (a class C gene) to regulate ovule development and floral meristem termination [159]. Loss-of-function in *OsMADS2*1 showed no ovule defects suggesting a loss of ovule specification by this gene [92,99]. 

Maize has three class D genes: *ZMM1*, *ZAG2*, and *ZMM2*5 [62]. Phylogenetic analysis showed *ZMM1* and *ZAG2* to be closely related to rice *OsMADS13*, whereas *ZMM2*5 had a close relationship with rice *OsMADS21* [131]. Similar to Arabidopsis *STK*, the expression of *ZAG2* was primarily identified in carpels and ovules [97,160] indicating a possible role in ovule specification.

Like rice, Brachypodium also has two D class genes—*BdMADS2* and *4*—orthologous to *OsMADS13* and *OsMADS21*, respectively. Quantitative RT-PCR revealed comparable expressions of both genes in all floral organs, with the exception of carpels and ovules, where the expression of *BdMADS2* was more than 5 times to that of *BdMADS4* [30]. Ectopic expression of both genes in Arabidopsis demonstrated that overexpression of *BdMADS4* produced more significant phenotypic changes than transgenic Arabidopsis carrying *BdMADS2*. Interestingly, in contrast to Arabidopsis and rice D class genes, overexpression of Brachypodium D-lineage genes did not directly affect carpel and ovule development in transgenic Arabidopsis. Further studies involving loss-of-function mutants would be required to confirm their role in ovule identity.

Wheat *SEEDSTICK* (*WSTK*) is an ortholog of Arabidopsis *STK* and rice *OsMADS13.* In wheat, its homologous genes are identified as *TaAGL9* and *TaAGL31* [117]. *WSTK* expression was observed in young to mature spikes, although its transcription was only restricted to pistils. During ovule development, the highest expression of *WSTK* was observed in the developing ovule of the pistils, suggesting an involvement in ovule specification and development [90]. Moreover, *WSTK* was expressed not only in true pistils but also in pistil-like stamens of an alloplasmic wheat line having the *Aegilops crassa* Boiss. cytoplasm, which arose due to the homeotic transformation of stamens into pistil-like structures. During the homeotic transformation of stamens into pistil-like structures in an alloplasmic wheat line no significant difference were recorded in the expression of wheat class C gene homologs *WAG-1* and *WAG-2* [161]. Furthermore, yeast two-hybrid analysis demonstrated that the WSTK protein formed a complex with a class E protein (WSEP) [90]. These observations suggest that similar to Arabidopsi*s* STK, wheat STK protein interacts with the E class protein to specify ovule identity providing an evidence functional conservation of class D genes in Arabidopsis and wheat. 

### 3.4. Class E Homeotic Genes

Class E genes work in all floral organs and act as cofactors for A, B, C, and D class proteins to form higher order MADS-box protein complexes, which regulate the floral organ identity (floral quartet model) [162]. In Arabidopsis, four *SEPALLATA* genes (*SEP1*-*4*) have been reported and these specify sepal, petal, stamen, carpel, and ovule identity [34,35]. Knockdown of all of these genes results in the transformation of floral organs into bract-like structures and sepals. 

In grasses, *SEP*-like genes are further classified into *SEP* and *LOFSEP* clades and *AGL6*-like genes [114,115,163]. The *SEP* clade in rice include *OsMADS7* and *OsMADS8.* Cosuppression of both genes results in severe homeotic and meristematic changes in all floral organs, especially in lodicules [104]. *OsMADS1*/*LEAFY HULL STERILE1* (*LHS1*), *OsMADS5*, and *OsMADS3*4/*PANICLE PHYTOMER 2* (*PAP2*) are placed into the *LOFSEP* clade [110]. Mutations in *OsMADS1* produced an abnormal phenotype, which is described by the presence of lemma/palea-like leaves and lodicules [59]. Loss of *OsMADS1* transforms the lemma into glume-like structures [164]. Simultaneous knockdown of *OsMADS1*, *OsMADS5*, *OsMADS7*, and *OsMADS8* transforms the inner floral whorls into bract-like structures with no effect on the lemma [104] suggesting that *OsMADS1* is associated with lemma and palea differentiation. A more recent study confirms that *OsMADS1* is involved in floral meristem identity and activity because defective floral organs were observed in the outer two whorls of *osmads-1* flowers [165]. Yeast two-hybrid analysis showed OsMADS1 protein to form heterodimers with B, C and, D class proteins that modulate floral meristem determinacy and organ identity. *OsMADS1* and *OsMADS13* regulate meristem determinacy in partially independent pathways while *OsMADS17* is a direct target of *OsMADS1* during floral development. *OsMADS1* interacts physically and genetically with *OsMADS3* and *OsMADS58* to specify stamen identity and suppression of spikelet meristem reversion [165]. These findings suggest that *OsMADS1*, through physical and genetic interaction with floral homeotic regulators, has diversified functions in floral meristem maintenance and specification of organ identity. In contrast, mutations in *OsMADS34* disturb inflorescence morphology and interfere with primary and secondary branches [111]. *OsMADS34* has been shown to interact with rice class (A)-genes to define inflorescence meristem identity [112]. These observations demonstrate that *OsMADS34* plays an important role in inflorescence and spikelet meristem determination. Ren et al. [166] recently demonstrated that a new mutant allele (*m34-z*) of *OsMADS34* homeotically converted empty glumes into lemma-like organs, suggesting that *OsMADS34* is required for glume specification.

The rice *AGL6* clade contains two genes: *OsMADS6*/*MOSAIC FLORAL ORGANS 1* (*MFO1*) and *OsMADS17* [114,115]. Both genes specify floral organ identity, although the predominant role is played by *MFO1*. Mutant analysis showed that *MFO1* determines floral organs by synergistically interacting with all classes of homeotic genes, except class-(A) [113]. Interestingly, a null allele of *MFO1* converts all floral organs into lemma like structures, except the lemma, suggesting a critical role for *MFO1* in floral organ specification [167]. 

At least ten putative E class genes have been identified in maize, which can be further subcategorized into *SEP*, *LOFSEP*, and *AGL6* clades [105,109]. The *SEP* clade contains three genes (*ZMM6*, *7*, and *27*). The *LOFSEP* clade contains five genes (*ZMM3*, *8*, *14*, *24*, and *31*) and the *AGL6* clade contains two genes (*ZAG3* and *5*). Sequence and phylogenetic analysis showed that *ZMM3* was orthologous to *OsMADS5*, *ZMM6* was orthologous to *OsMADS7*, *ZMM7* and *ZMM27* were orthologous to *OsMADS8*, *ZMM24* and *ZMM31* were orthologous to *PAP2*/*OsMADS34*, and *ZMM8* and *ZMM14* were orthologous to *LHS1*/*OsMADS1* (Figure 3). During spikelet development, *ZMM8* and *ZMM14* were expressed in upper florets, although not in the lower florets of floral organs [109], indicating a possible role in floral meristem determinacy. In situ hybridization showed that *ZMM6* and *ZMM27* were strongly expressed during the maize kernel development, with lower expression during inflorescence development and no expression at all during vegetative growth [105]. Furthermore, neither single nor double knockdown mutants of *ZMM6* and/or *ZMM27* resulted in kernel abnormalities or alterations in flower development indicating functional redundancy of class E genes in maize.

Similar to rice and maize, Brachypodium also contains six cass-E genes—*BdMADS1*, *7*, *11*, *26*, *28*, and *32*—which are further classified into *SEP*, *LOFSEP*, and *AGL6* clades [31]. The *SEP* clade contains two genes—*BdMADS26* and *32*—both of which expressed highly in all floral organs except for the lemma and palea. The *LOFSEP* clade contains *BdMADS1*, *7*, and *11*. Strong expression of *BdMADS7* and *BdMADS11* was detected in all floral organs. On the other hand, the *AGL6* clade contains only one gene—*BdMADS28*—which was weakly expressed in lodicules and stamens only [31]. These diversified expression patterns are consistent with those of rice and maize homologs and indicate functional divergence among different class E clades. To date, none of the *SEP* encoding genes has yet been functionally characterized in Brachypodium. 

Wheat E class genes are also subcategorized into *SEP*, *LOFSEP*, and *AGL6* clades. The *SEP* clade contains wheat *SEP* (*WSEP*), *TaMADS1*, *TaAGL16*, *TaAGL28*, and *TaAGL30* genes [107,117]. In situ expression analysis showed *WSEP* in lodicules, stamens, and carpels during floral organ differentiation. Stronger expression of *WSEP* was observed in the palea after determination of floral organ identity, supporting the concept that in addition to organ differentiation, *WSEP* has a role in subsequent development [107]. Rice *AGL6* clade gene *OsMADS6* also showed palea specific expression [116]. Similar to Arabidopsis *SEP3*, *WSEP* also interacts with class B and C homeotic genes, suggesting a conserved role for grass E genes. *TaMADS1* is another *SEP* encoding gene that is characterized as an E class gene [108] and orthologous to the rice class E gene *OsMADS8/24*. *TaMADS1* is functionally similar to *WSEP* in that overexpression of both genes in transgenic Arabidopsis caused early flowering and terminal flower formation [107].

The *LOFSEP* clade in wheat contains eight genes: *WLHS1*, *TaAGL3*, *5*, *8*, *24*, *27*, *34*, and *40*. *LEAFY HULL STERILE 1* (*WLHS1*) is an ortholog of *OsMADS1/LHS1* [107,117]. High transcript levels of *WLHS1* accumulate in glumes, lemma, palea, and lodicules while stamens and pistils exhibiting low levels. This differential expression behavior of *LHS1* was also reported in other grass species including *Avena sativa*, *Chasmanthium latifolium*, *Pennisetum glaucum*, and *Sorghum bicolor* [168], which may be due to differences in corresponding inflorescence structures. The wheat *AGL6* clade contains two genes: *TaAGL6* and *TaMADS37* [117]. Mutants of the wheat *LOFSEP* and *AGL6* clades have not been identified and thus their function is unknown. 

## 4. Non MADS-Box Genes Involved In Floral Organ Identity

Some non-MADS-box genes are also reported to regulate floral development. Mutants of rice *aberrant panicle organization1* (*apo1*) showed phenotypic resemblance with class C gene mutants. *APO1* mutants convert stamens into lodicules with extra carpels, suggesting that *APO1* positively regulates class C gene functions [121,122]. Moreover, expression of the rice class C gene (*OsMADS3*) was reduced in *apo1* mutants indicating that *APO1* positively regulates *OsMADS3* expression [169]. The Arabidopsis genes *UNUSUAL FLORAL ORGANS (UFO)* and *APO1* are orthologs and both encode F-box proteins. *UFO* activates class B genes [123], suggesting a distinct role for both genes irrespective of their similar biochemical functions. Arabidopsis *FLORICAULA* (*FLO*)*/LEAFY* (*LFY*) and its rice ortholog *RICE FLORICAULA/LEAFY* (*RFL*)*/APO2* displayed different yet overlapping functions. For example, *RFL/APO2* specifies inflorescence meristem identity through interaction with *APO1* [170], whereas *FLO/LFY* specifies floral meristem identity and activates class A, B, and C genes [171].

As described above, carpel identity is defined by class C MADS-box genes, however *YABBY* TFs are also reported to play a major role in carpel identity. The rice mutant *drooping leaf (dl)* has some functional similarity to class C MADS TFs in specifying the carpel identity and mutation in *DL* converts carpels into stamens [80]. These findings support the notion that candidate carpel identity genes in rice (class C and *DL*) redundantly regulate class C gene functions. *DL* and the class B gene *OsMADS16/SPW1* antagonize each other and this antagonism is critical to setting boundaries between stamen and carpel identity [80]. The Arabidopsis gene *CRABSCLAW (CRC)* and rice *DL* both encode *YABBY* TFs, although in addition to its function in carpel identity; *CRC* also has a role in nectary development [118]. The expression pattern of *CRC* in homeotic mutants suggests negative regulation by class-(A) and B genes. The wheat *DL* ortholog (*TaDL*), which was identified by homology screening [119] and expression in alloplasmic wheat, was found in true pistils as well as pistil-like stamens, suggesting its role in carpel specification. Moreover, class B genes were not expressed in pistil like stamens indicating that *TaDL* and class B genes are mutually antagonistic [77]. Like rice and wheat, the maize *DL* mutants (*drl1* and *drl2*) have been characterized and cloned. The *drl* mutants displayed ectopic inner-whorl organs in pistillate and staminate florets [120]. Although meristem activity was influenced by the expression of the *Drl* loci, *Drl* transcripts were absent in floral meristems suggesting that *Drl* genes may function autonomously. Sang et al. [125] characterised *CHIMERIC FLORAL ORGANS 1 (CFO1)*, a MADS-box gene, which regulates floral organ identity in rice. Mutants of *CFO1* showed disrupted marginal palea with ectopic but chimeric floral organs. Expression pattern analysis revealed that rice *DL* was ectopically expressed in defective floral organs of *cfo1* flowers, suggesting negative regulation between *CFO1* and *DL* [125].

More recently, Liu et al. [124] reported that *LONG STERILE LEMMA1 (G1)/ELONGATED EMPTY GLUME (ELE)* and *OsMADS34/PAP2* were associated with rice lemma development and determination of empty glume identity. Mutants of *G1/ELE* showed homeotic transformation of empty glumes into lemma like organs. Single and double mutants of *G1/ELE* and *OsMADS1/OsLHS1* showed redundant roles for both genes in controlling empty glume identity and lemma development. Expression analysis of *G1/ELE* in *osmads-1* flowers and *OsMADS1/LHS1* in *g-1* flowers indicated that both genes are regulated through independent pathways and do not interact at the transcription level. In *G1/ELE* mutant plants, downregulation of empty glume identity genes and ectopic expression of lemma identity genes provides strong evidence that empty glumes are in-fact sterile lemmas. Moreover, Yang et al. [126] identified a single recessive gene *lemma-distortion1 (ld1)* associated with lemma development in rice. This gene encodes a zinc finger protein. Overall these reports suggest that a plethora of non-MADS-box genes are also involved in floral organ identity in eudicots and cereals. 

## 5. Functional Conservation and Diversification between Distinct Floral Specification Systems

If it is assumed that all angiosperms have homologous reproductive organs, then divergent angiosperm groups may have a single ancestral flower specifying genetic mechanism. The modified floral organ identity model (ABCDE) in Arabidopsis suggests occurrence of genetic interactions among floral homeotic genes. The same model can be used to interpret molecular control of inflorescence identity in other crop plants including cereals [37]. High-throughput forward and reverse genetic approaches have led to the identification, cloning, and functional characterization of several genes involved in the regulation of floral development especially in grasses. Interestingly, most of these genes exhibit highest sequence similarites and share expression patterns and functional properties with those of eudicot A, B, C, D, and E floral homeotic genes. However, some grass-specific floral regulators have also been identified that do not have eudicot homologs and perform distinct functions in grass floral development. This review integrates current knowledge of floral organ identity genes in an attempt to adopt the eudicot floral organ identity model to other crop species. Considering grass organ-identity models illustrated in Figure 2, it is apparent that further research is needed to functionally characterize maize, Brachypodium, and wheat MADS-box genes to manipulate for crop grain yield improvement. 

Prior to the discovery of loss-of-function mutants, gene function was usually examined through sequence conservation and expression pattern comparisons with already characterised genes. Due to genome complexities and difficulties in the use of modern genetic approaches in grasses, very little was known about the role of MADS-box genes in controlling spikelet and floret development. However, recent studies have provided new insights into the conservation of class (A)-gene function among eudicots and cereals [65]. The mutational analyses of *AP1/FUL* like genes in rice and demonstrated that in addition to their role in floral meristem identity, they also influenced the specification of palea and lodicule identities. As these grass-specific organs are thought to be homologous to eudicot’s sepals and petals, these divergent groups may share a conserved nonreproductive floral organ specification system.

Comparison of all the proposed models indicates a partially conservative, partially-diverse floral regulation among grasses and higher eudicots. Based on studies in model eudicots, gene expression patterns and mutant phenotypes appear to be consistent with functional predictions. This is essentially true for class B, C, and D MADS-box genes. Like eudicots, the functions of class B and C genes have diverged in grasses due to duplication and subfunctionalization of separate genes. For example, rice *PI*-like class B genes show unequal redundancy in their function. Individual mutant analysis of *OsMADS2* indicates the homeotic conversion of lodicules without affecting stamens, whereas *OsMADS4* shows no alteration in lodicules and stamens. Additionally, double mutants of both genes show the conversion of lodicule and stamen into palea and carpel-like structures, suggesting an equal role of both genes in stamen identity. However, *OsMADS2* is more important than *OsMADS4* in lodicule identity. Mutant analysis of maize *PI* orthologs also indicated that *ZMM16/STS1* can compensate *zmm18/29* reduction while specifying class B gene functions. However, *ZMM18/29* cannot compensate for *zmm16/sts1* reduction. Similar roles may be speculated for Brachypodium and wheat *PI* orthologs as *BdMADS16* and *WPI1* show sequence similarity with *OsMADS4* and *ZMM18*, respectively, while *BdMADS20* and *WPI2* have greater similarity with respect to *OsMADS2* and *ZMM16* (Figure 3).

Subfunctionalization of duplicated genes was also observed among grass related class C genes. According to Yamaguchi et al. [98], *OsMADS58* plays a major role in floral meristem determinacy with minor effects on floral organ identity; whereas *OsMADS3* has a dominant role in stamen identity with a minor role in meristem determinacy. Similar findings were reported by Dreni et al. [92], in which *OsMADS3* showed to regulate stamen identity compared with *OsMADS58*. Severe defects were obsereved in *osmads3* mutant flowers, whereas most of the *osmads58* mutant flowers were indistinguishable from the wild type flowers. Likewise, the maize orthologs *ZAG1* and *ZMM2* exhibit functional diversification and are homologs to *OsMADS58* and *OsMADS3*, respectively. *ZAG1* mutant showed loss of floral meristem determinacy and have a minor role in stamen and carpel identity. By contrast, *ZMM2* has yet to be characterized for floral organ identity. In Brachypodium, *BdMADS14* and *BdMADS18* also indicated overlapping but diverse expression patterns as *BdMADS14* highly expressed in stamens only compared to *BdMADS18* that strongly expressed both in stamens and carpels. Based on orthologous relationships, Brachypodium and maize class C genes have overlapping but nonidentical functions, similar to rice genes. In contrast, the wheat class C genes *WAG-1* and *WAG-2* are involved in ectopic ovule formation and homeotic conversion of stamens into pistil- like structures and are orthologous to *OsMADS58* and *OsMADS3,* respectively. Knockdown mutants of *WAG* genes will help elucidate their function in floral organ identity. Mutations in rice and maize class C genes result in homeotic conversion of stamens and carpels into lodicules and paleae-like organs, respectively. Similarly, class C gene mutations in Arabidopsis caused homeotic conversion of both reproductive organs, with few exceptions. Mutations of these genes in wheat result in ectopic ovule formation and homeotic conversion of stamens into pistil-like structures. Thus, diverse carpel specification systems operate in these two divergent groups. 

Genetic interactions between rice class C and D MADS-box and non-MADS-box (*DL*) genes provided new insights into the partially conservative and partially diversified mechanisms regulating floral development in eudicots and grasses. Li et al. [159] studied double mutants of *OsMADS3*, *OsMADS13*, and *DROOPING LEAF* (*DL*) to investigate their role in floral development. Termination of floral meristem determinacy and several carpelloid structures were observed in *osmads3/osmads13* double mutants, while noteably their single mutants lacked these alterations at all. Furthermore, gene expression and protein–protein interaction analyses revealed that both C and D class genes neither regulate nor interact at the transcription or protein levels, suggesting that class E genes would mediate their interaction to synergistically control the termination of floral meristem and ovule identity. These obervations support the notion that grasses have retained their class C and D gene functions, despite undergone duplication and subfunctionalization. Dramatically, double mutants of *osmads3/dl* showed no *AG* activity, with production of lodicule-like structures within the fourth whorl and the termination of floral meristem determinacy. Mutual suppression was also absent and normal expression patterns were observed for *OsMADS3* and *DL* genes in ectopic stamens of *dl* flowers and *osmads3* mutants, respectively, suggesting a redundant role for both genes in floral meristem termination. In contrast, single and double mutants of *OsMADS13* and *DL* suggest that *DL* is epistatic to *OsMADS3*, and both have identical roles in ovule identity. More recently, the role of ovule in cereal grain development has been briefly reviewd [172]. The phenotype of single *dl* mutants was identical to that of double *osmads13/dl* mutants. The *dl* mutants lacked *OsMADS13* expression, whereas single mutant of *osmads13* showed abundance of *DL* transcripts, indicating direct or indirect *OsMADS13* regulation by *DL*. Dreni et al. [92] investigated genetic interactions between rice class C and D genes (*OsMADS13* and *OsMADS58*) through single- and multiple-gene mutants. As expected, *osmads13/58* double mutants showed accumulation of lodicule and palea-like organs in the third and fourth whorls accompanied by loss of floral organ identity and the triggering of floral meristem indeterminacy due to reduced *AG* activity. These observations suggest that a highly conserved class C gene functional mechanism exists in grasses despite partial subfunctionalization among duplicated genes. These results led to the proposition that *DL* lacks class C gene functional activity and cannot specify carpel identity alone, requiring both *OsMADS3* and *OsMADS58*. Although these findings provide new insights into the floral development process, further examination of single and multiple mutants of class B, C, D, and *DL* genes will be required to elucidate the roles of MADS-box and *DL* genes in floral organ specification. 

The function of class E genes, particularly the *SEP*-like, are somewhat conserved among eudicots and grasses. In rice, detailed spatial and temporal mRNA expression studies, protein interaction patterns, and mutant analysis indicated a consistent role for *SEP*-like genes in floral meristem and organ identity specification [104], since divergence from eudicots. Mutant phenotypes of Arabidopsis *SEP* redundant genes (*SEP1/2/3/4*) and rice *OsMADS1*, *OsMADS7*, and *OsMADS8* genes indicated a redundant but interdependent role for both groups, suggesting a partial overlap but subfunctionalization among class E genes. Furthermore, characterization of *AGL6* clade mutants of rice and maize indicated a similar functional role in floral meristem determinacy and organ identity [114,115,159]. Similar expression patterns of monocot *AGL6* and *SEP* clade genes and their complex interactions with class B, C, and D genes indicated conserved floral specification systems. The functional similarity of *SEP* and *AGL6* clades is provided by Petunia floral development genes [173]. 

## 6. Future Perspectives

Understanding of grass inflorescence morphogenesis has expanded significantly over the last two decades. Extensive studies in model plants have demonstrated common genetic factors regulating eudicot and grass floral development including MADS-box and non-MADS-box genes and epigenetic regulators. For Arabidopsis and rice, the genetic and molecular mechanisms of transition from vegetative to reproductive phase and the role of MADS-box genes in floral organ identity are well understood. However, this is less well defined for Brachypodium, maize, and wheat because loss-of-function mutant analysis is rare in these species. Currently, advances in genetics analysis has made mutant development and characterization easy in grasses which is being used to define grass floral developmental biology. Deciphering the molecular control of transition from shoot apical meristem to floral meristem development and the determination of floral organ identity will provide new insights to devise innovative strategies for the development of cereals with enhanced grain yield and adaptation to multiple environments [174]. 

Biological research in general and plant evolutionary biology have been revolutionized by advances in next generation sequencing. Enormous amounts of genomic and transcriptomic sequence data have also been generated through the 1000 Plants Project (1KP) and the Floral Genome Project [175,176]. These gene resources provide an unprecedented opportunity to bridge the evolutionary gap between floral morphogenesis in model plants and economically important cereals by characterizing floral genetic components of ABCD model. Here, it is important to note that most of the cereal orthologs are merely retrieved by phylogenetic analysis from resource genome databases; therefore, specified experimental studies will be required to support genetic framework of underlying mechanisms of floral organ identity in cereals. In this perspective, genome editing tools such as virus-induced gene silencing (VIGS) and clustered regularly interspaced short palindromic repeats (CRISPR)/Cas9 system has been widely useful in several eudicots and monocot species to investigate gene function in floral organ identity and symmetry between basal and core eudicots [177,178,179,180,181]. Effective application of these systems could herald a new generation of multidisciplinary evo–devo research that better describes the evolutionary changes in gene regulatory networks underlying floral development. Moreover, these approaches along with TILLING resources can provide new avenues for grain yield improvement in cereals through translational research. 

## Figures and Tables

**Table 1:** Genes controlling floral organ identity in cereals.

Arabidopsis Gene	Subfamily	Class	Putative Role	Rice	Maize	Wheat	Brachypodium	References
*AP1*	*AP1*	A	Promote floral meristems, sepals, petals or lemma/palea identities	*OsMADS14*	*ZMM4, 15*	*WFUL1, TaAGL25*	*BrMADS33*	[31,58,59,60,61,62,63,64,65,66]
				*OsMADS15*	*ZAP1*	*WFUL2*	*BrMADS10*	
				*OsMADS18*	*-*	*WFUL3, TaAGL10*	*BrMADS3*	
				*OsMADS20*	*-*	*-*	*BrMADS31*	
*AP2*	*AP2*	A	Spikelet/floral meristem identity and lodicule identity	*IDS1*	*ids1*	*TaAP2*	*-*	[24,25,67,68,69,70,71,72,73,74,75]
				*SNB*	*sid1*	*TaQ*	*-*	
				*MFS1*	*-*	*-*	*-*	
				*FZP*	*BD1*	*WFZP*	*MOS1*	
*AP3*	*AP3*	B	Lodicule and stamen identity	*OsMADS16/SPW1*	*Silky1*	*WAP3*	*BrMADS5*	[31,76,77,78,79,80]
*PI*	*PI*	B		*OsMADS2*	*ZMM16*	*WPI2*	*BrMADS20*	[31,77,81,82,83,84,85,86]
				*OsMADS4*	*ZMM18, 29*	*WPI1*	*BrMADS16*	
*ABS/TT16, GOA*	*B_SISTER_*	-	Integuments and seed development	*OsMADS29, 30*	*ZMM17*	*WB_sis_, TaAGL35*	*BrMADS17, 23, 38*	[31,84,87,88,89,90,91]
*AG, SHP1, SHP2*	*AG*	C	Stamen and carpel identity	*OsMADS3*	*ZMM2*	*WAG-2*	*BrMADS14*	[31,57,92,93,94,95,96,97,98]
				*OsMADS58*	*ZAG1*	*WAG-1*	*BrMADS18*	
*STK*	*AG*	D	Ovule identity	*OsMADS13*	*ZMM1, ZAG2*	*WSTK, TaAGL9, 31*	*BrMADS2*	[30,31,57,62,90,99,100,101,102,103]
				*OsMADS21*	*-*	*-*	*BrMADS4*	
*SEP (1-4)*	*SEP*	E	FM determinacy and floral organ identity	*OsMADS7/45*	*ZMM6*	*WSEP, TaAGL16, 28,30*	*BrMADS26*	[31,34,104,105,106,107,108]
				*OsMADS8/24*	*ZMM7/27*	*TaMADS1*	*BrMADS32*	
-	*LOFSEP*	E		*OsMADS1/LHS1*	*ZMM8/14*	*WLHS1, TaAGL24*	*BrMADS11*	[31,104,107,109,110,111,112]
				*OsMADS5*	*-*	*TaAGL3, 5, 8, 34, 40*	*BrMADS7*	
				*OsMADS34/PAP2*	*ZMM24, 31*	*TaAGL27*	*BrMADS1*	
-	*AGL6*	E		*OsMADS6/MFO1*	*ZAG3, 5*	*TaAGL6, 37*	*BrMADS28*	[31,82,105,113,114,115,116,117]
				*OsMADS17*	*-*	*-*	*-*	
*CRC*	*YABBY*-like	-	Carpel identity	*DL*	*Drl1, 2*	*TaDL*	*-*	[77,80,118,119,120]
*UFO*	F-box	-	Positive regulation of B & C class MADS	*APO1*	*-*	*-*	*-*	[121,122,123]
*-*	DoF	-	Lemma and Palea identity?	*CFO1, G1/ELE, ld-1*	*-*	*-*	*-*	[124,125,126]

## Supplementary Materials

Supplementary materials can be found at https://www.mdpi.com/1422-0067/20/11/2743/s1.

## Abbreviations

*ABS*	*ARABIDOPSIS BSISTER*
*AG*	*AGAMOUS*
*AGL6*	*AGAMOUS-LIKE 6*
*AP1*	*APETALA1*
*AP2*	*APETALA2*
*AP3*	*APETALA3*
*APO1*	*Aberrant panicle organization-1*
*BD1*	*BRANCHED SILKLESS1*
*BdMADS*	*Brachypodium MADS*
BM	Branch meristem
BR	Branch
CA	Carpel
*CAL*	*CAULIFLOWER*
*CFO1*	*CHIMERIC FLORAL ORGANS 1*
*CRC*	*CRABSCLAW*
*DL*	*Drooping leaf*
*ELE*	*ELONGATED EMPTY GLUME*
*FBP11*	*FLORAL BINDINGPROTEIN 11*
*FBP7*	*FLORAL BINDINGPROTEIN 7*
*FLO/LFY*	*FLORICAULA/ LEAFY*
FM	Floral meristem
*FUL1*	*FRUITFULL 1*
*FUL2*	*FRUITFULL 2*
*FUL3*	*FRUITFULL 3*
*FZP*	*FRIZZY PANICLE*
*G1*	*LONG STERILE LEMMA1*
GL	Glume
*GOA*	*GORDITA*
*HvAP2/Cly1*	*Hordeum vulgare APETALA2/Cleistogamy1*
*IDS1*	*INDETERMINATE SPIKELET1*
IM	Inflorescence meristem
*ld-1*	*lemma-distortion 1*
Le	Lemma
*LHS1*	*LEAFY HULL STERILE 1*
LO	Lodicule
LS	Lateral spikelet
LSM	Lateral spikelet meristem
*MADS*	*MINICHROMOSOME MAINTENANCE 1, AGAMOUS, DEFICIENS, and SERUM RESPONSE FACTOR*
MEGA6	Molecular Evolutionary Genetic Analysis version 6
*MFO1*	*MOSAIC FLORAL ORGANS 1*
*MFS1*	*MULTI-FLORET SPIKELET 1*
miRNA	micro-RNA
*MOS1*	*MORE SPIKELET 1*
mRNA	Messenger RNA
NCBI	National Center for Biotechnology Information
*OsIDS1*	*Oryza sativa INDETERMINATE SPIKELET 1*
*OsMADS*	*Oryza sative MADS*
OV	Ovule
PA	Palea
*PAP2*	*PANICLE PHYTOMER2*
PB	Primary branch
PBM/pBM	Primary branch meristem
PE	Petal
*PI*	*PISTILLATA*
PI	Pistil
RA	Rachis
Ra	Rachilla
*RFL*	*RICE FLORICAULA*
RNAi	RNA interference
RT-PCR	Reverse transcriptase-polymerase chain reaction
SAM	Shoot apical meristem
SB	Secondary branch
SBM/sBM	Secondary branch meristem
*SBP/SPL*	*SQUAMOSA PROMOTER BINDING PROTEIN-LIKE*
SE	Sepal
*SEP*	*SEPALLATA*
*SHP*	*SHATTERPROOF*
*SID1*	*SISTER OF INDETERMINATE SPIKELET1*
*Sl1*	*SILKY1*
SM	Spikelet meristem
*SNB*	*SUPERNUMERARY BRACT*
SPM	Spikelet pair meristem
*SPW1*	*SUPERWOMAN1*
ST	Stamen
STK	SEEDSTICK
*STS1*	*Sterile Tassel Silky Ear1*
*TaAG3*	*Triticum aestivum AGAMOUS3*
*TaAP3*	*Triticum aestivum AP3*
*TaDL*	*Triticum aestivum Drooping Leaf*
*TaMADS*	*Triticum aestivum MADS*
TFs	Transcription factors
TS	Terminal spikelet
TSM	Terminal spikelet meristem
*TT16*	*TRANSPARENT TESTA 16*
*UFO*	*UNUSUAL FLORAL ORGANS*
*VRN1*	*VERNALIZATION 1*
*WAG-1*	*Wheat AGAMOUS-1*
*WAG-2*	*Wheat AGAMOUS-2*
*WAP1*	*Wheat APETALA1*
*WAP3*	*Wheat APETALA3*
*WBsis*	*Wheat BSISTER*
*WFUL1*	*Wheat FRUITFULL 1*
*WFUL2*	*Wheat FRUITFULL 2*
*WFUL3*	*Wheat FRUITFULL 3*
*WLHS1*	*Wheat LEAFY HULL STERILE 1*
*WPl1*	*Wheat PISTILLATA 1*
*WPI2*	*Wheat PISTILLATA 2*
*WSEP*	*Wheat SEPALLATA*
*WSTK*	*Wheat SEEDSTICK*
*ZAG1*	*Zea mays AGAMOUS1*
*ZAG2*	*Zea mays AGAMOUS2*
*ZAP1*	*Zea mays APETALA1*
*ZmIDS1*	*Zea mays INDETERMINATE SPIKELET1*
*ZMM*	*Zea mays MADS*
